# ﻿Taxonomic revision of the genus *Phylacastus* Fairmaire (Tenebrionidae, Eurynotina): shortfalls of anatomical nomenclature with notes on aedeagal homology

**DOI:** 10.3897/zookeys.1138.95968

**Published:** 2023-01-05

**Authors:** Ryan Lumen, Marcin Jan Kamiński

**Affiliations:** 1 Zoological Museum, Museum and Institute of Zoology, Polish Academy of Sciences, Warsaw, Poland Zoological Museum, Museum and Institute of Zoology, Polish Academy of Sciences Warsaw Poland; 2 Department of Entomology, Purdue University, West Lafayette, Indiana, USA Purdue University West Lafayette United States of America

**Keywords:** Amphidorini, clavae, Dendarini, laciniae, median lobe, parameres, Pedinini, South Africa

## Abstract

The genus *Phylacastus* Fairmaire (Tenebrionidae, Blaptinae, Platynotini, Eurynotina) is revised. Two new species and one new synonymy are presented along with new diagnoses, descriptions, a distribution map, and key to species. The resulting species of *Phylacastus* are: *P.ancoralium***sp. nov.**, *P.crypticoides* Koch (= *P.pretoriensis* Koch **syn. nov.**), *P.makskacymirowi***sp. nov.**, *P.rhodesianus* Koch, and *P.striolatus* Fairmaire. Lectotypes are designated for the type species, *P.striolatus*, to fix the taxonomic status of the species and genus. As a result of examination and subsequent description of *P.ancoralium***sp. nov.**, a brief review and treatment of aedeagal morphology is presented. The nomenclature (“clavae” versus “laciniae”) and phylogenetic occurrence of accessory structures of the paramere-median lobe area within Blaptinae Leach and Adelinina LeConte (Diaperinae, Diaperini) are discussed. New descriptive terminology (i.e., ancora [singular] and ancorae [plural]) is proposed for these aedeagal structures in Blaptinae to clarify their function and resolve past ambiguities. The morphology within representatives of *Adelina* Dejean, *Alphitophagus* Stephens, *Gnatocerus* Thunberg, and *Sitophagus* Mulsant is also briefly contrasted and outlined.

## ﻿Introduction

Eurynotina Mulsant & Rey is a subtribe of darkling beetles from Southern Africa within the tribe Platynotini Mulsant & Rey and subfamily Blaptinae Leach (Koch 1954a; Bouchard et al. 2021; Kamiński et al. 2021a). Platynotini are distinguished via the presence of a stridulatory file on the gula (synapomorphy for the tribe; see Koch 1954a, b, 1956). Eurynotina are further diagnosable via their aedeagi, which lack additional “styles”, “clavae”, or “lacinia” (Antoine 1930; Koch 1954a, b; Lindroth and Palmen 1956) and have a strongly sclerotized medial lobe with reduced basal apophyses (Iwan 2001). Eurynotina has been supported as molecularly distinct by Kamiński et al. (2019, 2021a); however, the taxa included were not fully sufficient to test the monophyly of the group. This paper is the first of a series dedicated to revising subtribe Eurynotina as a part of the first author’s Ph.D. dissertation.

Platynotini has received attention from many generations of entomologists (Fairmaire 1897; Gebien 1904, 1910; Reichardt 1936; Español 1945; Koch 1956, 1958; Kaszab 1975; Iwan 1995, 2002, 2006; Endrödy-Younga 2000; Kamiński and Raś 2011; Iwan and Kamiński 2012, 2014; Kamiński 2013, 2015a); however, most contributions concern the subtribe Platynotina Mulsant & Rey. Only a handful of papers concern Eurynotina (Koch 1954a, b, 1955, 1956; Kamiński 2016). For example, *Phylacastus* was erected in Opatrini Brullé by Fairmaire (1897) with a single new species (*P.striolatus* Fairmaire) and remained unstudied for nearly 60 years. In 1954a, Koch described three additional species and assigned the genus to his recently installed subtribe Oncotina Koch, now interpreted as a synonym of Eurynotina (see Kamiński 2016). He hypothesized a relationship between *Phylacastus* and *Eurynotus* Kirby through the following characters: horizontally produced prosternal apophysis, median emargination of epistoma, sharp and rectangular posterior angles of pronotum, and closely jointed prothorax and mesothorax. Prior to the study presented here, the only count of *Phylacastus* specimens was provided by Koch’s (1954a) work (34 specimens, 25 of which belonged to one of his new species *P.pretoriensis*, and two syntypes of *P.striolatus*).

After queries to several entomological collections (see list in Materials and Methods) we identified new specimens and species of the genus. These materials provided the opportunity to test the taxonomic concepts of *Phylacastus* and its species. Furthermore, as one of the newly discovered species challenges Koch’s (1954a, b) subtribal definition of Eurynotina, male terminalia morphology within subfamily Blaptinae is discussed based on dissected specimens, alongside previous literature (e.g. Koch 1956; Iwan 2001, 2004). Consequently, new terminology is proposed in light of previous application of the terms “clavae” and “laciniae” in the context of their meaning and priority within Blaptinae. They are also briefly contrasted with representatives of Diaperinae Latreille to better describe function, homology, and resolve some ambiguities.

## ﻿Materials and methods

### ﻿Revision of genus *Phylacastus*

Pinned material for morphological examination of *Phylacastus* and other taxa was borrowed from the following institutional insect collections: **MNHN** – Muséum national d’Histoire naturelle; Paris, France; and **TMNH** – Ditsong National Museum of Natural History; Pretoria, South Africa. Additional comparative material for redefining the genus and investigating aedeagal morphology was obtained from: **MIIZPAN** – Muzeum i Instytut Zoologii, Polska Akademia Nauk; Warsaw, Poland; **SANC** – South African National Collection of Insects; Pretoria, South Africa. While specimens of Eurynotina are relatively uncommon, the holdings of the aforementioned collections are the most comprehensive for the subtribe, accounting for both the majority of type material, and additional specimens for examination. As a result of specimen loans and contact with collections presented here, all 16 genera and over 90% of the species of Eurynotina are represented by type material and photographs for reference for this project and continued revision of the subtribe.

Original label data for specimens are given in quotation marks and separated by a comma. Morphological terminology follows that of Matthews at al. (2010), with additional specialized terms used for the female terminalia following Kamiński et al. (2022). Dissections were performed following methodology illustrated by Kamiński (2021); specimens were soaked in 10% KOH solution for dissection of genitalia before staining with chlorazol black. Images were taken using a Canon 1000D body with extension rings and a Canon EF 100 mm macro lens, a Nikon D3500 body with adapter for a Nikon SMZ800N microscope, and with a Hitachi S-3400N SEM in MIZ PAS. A species distribution map was produced using QGIS v. 3.16, with vector layers downloaded from the Natural Earth web page (www.naturalearthdata.com). Photographs as well as distribution map figures were edited in Photoshop v. 23.5.1. A table of all localities is presented in Appendix 1.

### ﻿Male terminalia analysis

Revelation of new structures on the aedeagus of *Phylacastusancoralium* sp. nov. necessitated a review of aedeagal morphology to confirm its affiliation. To this end, we performed a historical literature review, and assessed aedeagal terminology and morphology (Antoine 1930; Español 1945; Koch 1954a, b; Lindroth and Palmen 1956; Doyen and Tschinkel 1982; Doyen 1984; Iwan 2001, 2004; Kamiński 2014, 2015b). Taxon selection mainly focused on Blaptinae, as various subgroups have historically been defined by the presence or absence of additional structures of the parameres/median lobes (e.g. Platynotina and Eurynotina, Opatrini); however other groups of Tenebrionidae Latreille with structures described as “clavae”, “lacinia”, “struts”, or “styles” were also sampled for morphological study and comparison. Taxa were also chosen for potential homology and concurrent terminology based on literature descriptions. Taxa selected were: Blaptinae: *Amatodes* Dejean (Pedinini: Helopinina), *Anomalipus* Guérin-Méneville (Platynotini: Platynotina), *Eleodes* Eschscholtz (Amphidorini), *Heliopates* Dejean (Pedinini: Dendarina), *Trigonopus* Mulsant & Rey (Platynotini: Platynotina), and Diaperinae (Diaperini: Adelinina): *Adelina* Dejean, *Alphitophagus* Stephens, *Gnatocerus* Thunberg, and *Sitophagus* Mulsant.

## ﻿Taxonomy

### ﻿Genus Phylacastus

Taxon classificationAnimaliaColeopteraTenebrionidae

Fairmaire

1A2EA9FD-BFF2-5B4A-98F4-5EE28CF2A740

Phylacastus Fairmaire, 1897: 116. Koch 1954a: 275; 1954b: 2; 1956: 27; Kamiński 2016: 245.

#### Type species.

*Phylacastusstriolatus* Fairmaire; by monotypy.

#### Diagnosis.

**Figure 1.**
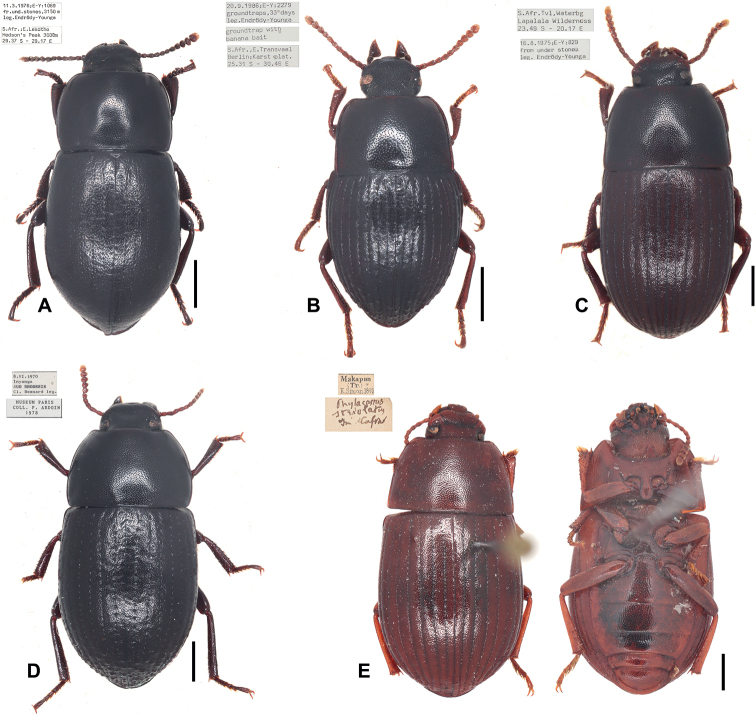
Dorsal habitus of *Phylacastus* species **A***Phylacastusancoralium* sp. nov. holotype **B***Phylacastusmakskacymirowi* sp. nov. **C***Phylacastuscrypticoides***D***Phylacastusrhodesianus***E***Phylacastusstriolatus* lectotype. Scale bars: 1 mm.

**Figure 2.**
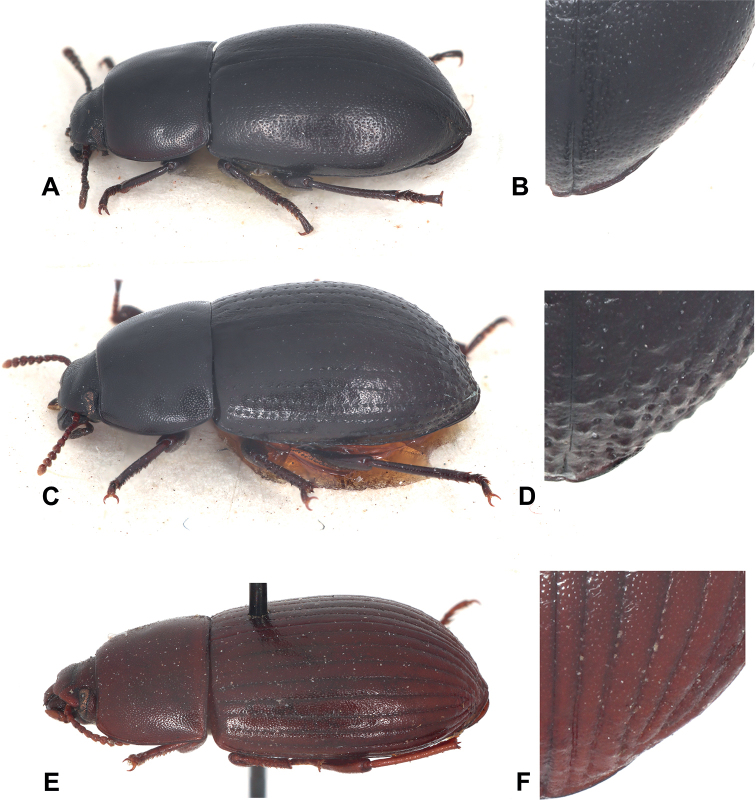
*Phylacastus* lateral aspect photographs and close-up of apical elytral tubercles and striae **A***Phylacastusancoralium* lateral angle **B***P.ancoralium* close-up of elytra apical declivity **C***P.rhodesianus* lateral angle **D***P.rhodesianus* close-up of elytra apical declivity **E***P.striolatus* lateral angle **F***P.striolatus* close-up of elytra apical declivity.

Within Eurynotina, *Phylacastus* largely resembles *Eurynotus* and *Capidium* Koch. All three have relatively sharp basal pronotal angles, rather than broadly rounded as is the case in the rest of Eurynotina (Kamiński 2016: fig. 2). The only other exception is *Oncotus* Solier which, while some representatives have basal angles of the pronotum similarly shaped, is separable by prosternal process shape (rounded rather than angular in lateral view (Kamiński 2016), body shape (much rounder/transverse than *Phylacastus*), tibial morphology (foretibia greatly expanded apically and with a sharp lateral projection; Kamiński 2016), and coloration (species may be bicolored and/or very pale or testaceous in color). *Phylacastus* can be easily separated from all other subtribal representatives by the presence of (at most) weak tubercles on the apical declivity of the elytra (Figs 1, 2), the form of the prosternal process which is angular rather than rounded in lateral view (Kamiński 2016: fig. 2D), and the pronotum with basal angles present rather than absent/rounded) (Kamiński 2016: fig. 2J).

**Figure 3.**
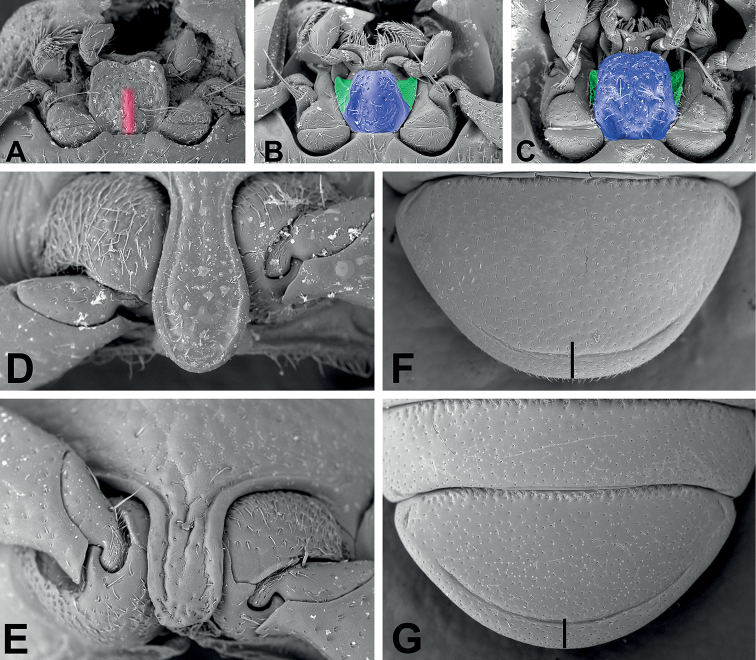
Diagnostic features of *Phylacastus* species **A–C** mentum (Median keel red, middle portion and lateral wings blue and green respectively) **D–E** prosternal process **F–G** abdominal ventrite V **A, D, F***Phylacastuscrypticoides***B***P.makskacymirowi***C***P.rhodesianus***E***P.ancoralium***G***P.striolatus*. Scale bars: 0.1 mm

*Eurynotus*, the most closely affiliated genus according to Koch (1954a), can be separated from *Phylacastus* by body size (*Eurynotus* ~9–20 mm long and ~5–12 mm wide, versus *Phylacastus* 4–8 mm long and ~2.75–4 mm wide (Koch 1954a; Kamiński 2016); pronotal hind angles (*Eurynotus* prominently produced often rearward projecting; less prominent and not rearwardly projected in *Phylacastus*; Kamiński 2016), tibial morphology (*Eurynotus* with slender/narrow tibiae lacking coarse spines on ventral surface of foretibia; dorsoventrally flattened and apically expanded tibiae with coarse spines on the underside of the foretibia in *Phylacastus* (Kamiński 2016), elytral sculpturing (*Eurynotus* with coarse or well-defined tubercles in most species; while most species of *Phylacastus* lack well-defined tubercles (Kamiński 2016). Finally, *Eurynotus* lacks a subapical sulcus on abdominal ventrite V, which is present in all *Phylacastus* species (Fig. 3F, G).

*Capidium* can be separated from *Phylacastus* most reliably via the structure of the prosternal process and abdominal ventrite V (prosternal process rounded and not produced in *Capidium*, angular and produced in *Phylacastus* (Kamiński 2016), and subapical sulcus absent in *Capidium* (present in *Phylacastus*); additionally, although *Capidium* also is defined by angular basal angles of the pronotum (Kamiński 2016), the angles are usually more produced. Finally, the elytral sculpturing and tuberculation of representatives of *Capidium* (when present) are stronger than in *Phylacastus*.

#### Genus redescription.

**Figure 4.**
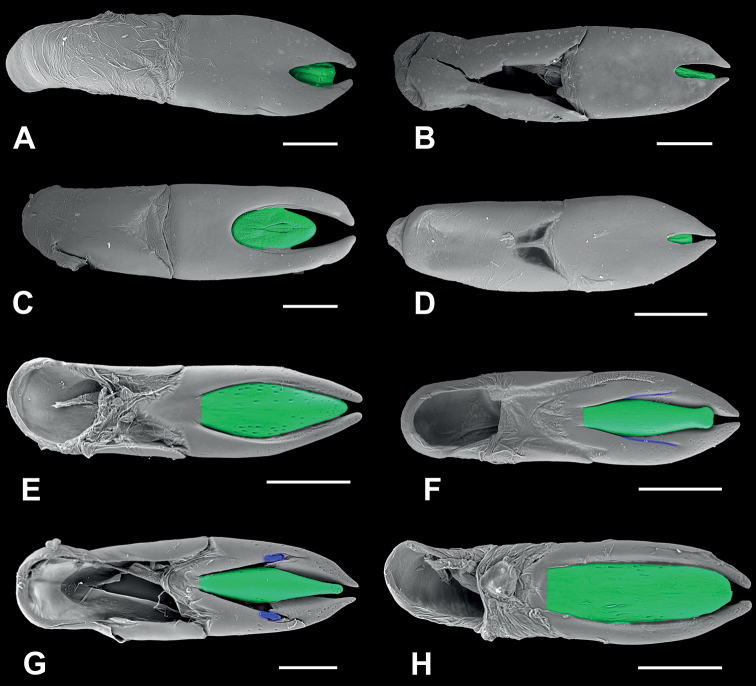
*Phylacastus species spp.* aedeagi **A–D** aedeagus dorsal view **E, F** aedeagus Ventral view **A, H***Phylacastusstriolatus***B, G***P.ancoralium* (ancorae highlighted blue) **C***P.rhodesianus***E***P.crypticoides***D, F***P.makskacymirowi* (subapical sutures highlighted blue). Median lobes highlighted green. Scale bars: 0.2 mm.

**Figure 5.**
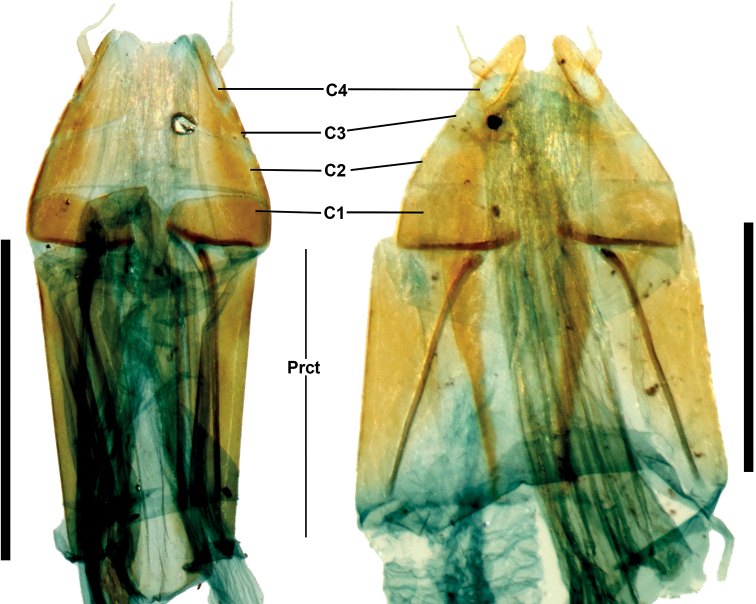
*Phylacastus* ovipositor (dorsal). **Right***Phylacastusancoralium***Left***P.crypticoides*. Abbreviations: C – Coxae (1–4); Prct – Paraprocts. Scale bars: 1 mm.

**Figure 6.**
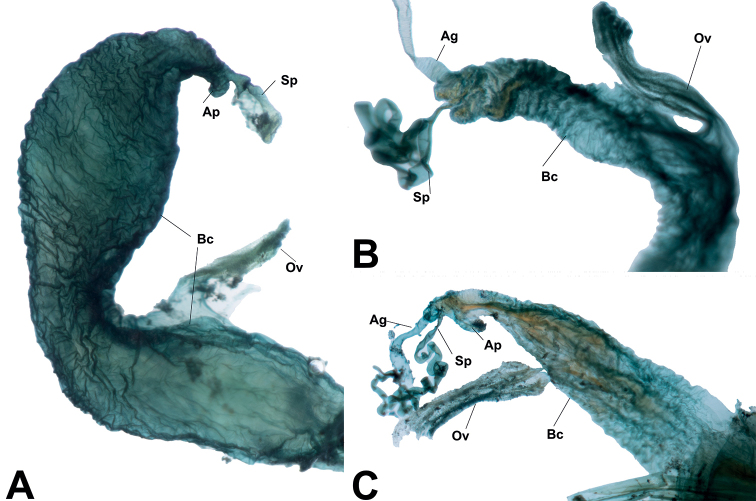
*Phylacastus* internal female structures **A***Phylacastusstriolatus***B***P.crypticoides***C***P.ancoralium*. Abbreviations: Ag - Accessory gland, Ap - Accessory pouch, Bc - Bursa copulatrix, Ov - Oviduct, *Sp* - Spermatheca.

Length 4–8 mm. Shining to dull; colored tenebrous; reddish to dark brown/black. ***Head***: epistoma with well-defined median notch. Transition between clypeus and frons gradual and smooth along lateral edge, or with slight depression. Coarsely punctate, punctures large and closely spaced, separated by ≤ 1 feature diameter. Mentum with enlarged, ventrally projecting middle portion parallel-sided to slightly narrowing apically with reduced/slightly hidden lateral wings. Gula with stridulatory file. Eye constricted in middle and reniform, with strong to weakly impressed sulcus situated around posterior perimeter of dorsal lobe. Antennae with 11 antennomeres, terminal members forming weak club. ***Prothorax***: pronotum base straight, with basal angles roundly produced. Without lateral depression or flattening along margins. Hypomeron at most only finely sculptured and finely punctured, dull to shining. Prosternal process angulate in lateral view, weakly produced or rounded at apex, with clear sulcus running perimeter, projecting at most only weakly toward midcoxae. ***Pterothorax***: scutellar shield small and transversely triangular. Elytra not costate, with or without shallow or weakly defined punctate striae. Intervals punctate, without microtubercles; weak to well-defined tubercles (when present) only on apical declivity. Interval X terminating before reaching elytra base. Epipleura without microtubercules, broad basally, narrowing apically. Apterous. ***Abdomen***: punctate. Ventrite V with sulcus running parallel to apical perimeter. ***Legs***: femora slightly curved and expanded toward apex. Tibiae dorsoventrally compressed. Meso- and metatibia slightly curved. Foretibia dilated triangularly toward apex with coarse spines underneath. ***Male terminalia***: tegmen bipartite with or without ancorae (small ancorae present in one species); basal portion membranous ventrally; dorsally with small, triangular membranous field at base of apical portion. Parameres fused dorsally at base, apical opening (in dorsal view) small or broad (Fig. 4). In lateral view, parameres flattened toward apex, with or without slight curvature. ***Female terminalia***: paraprocts nearly as long or slightly longer than coxites I–IV, coxite IV reflected dorsally with gonostyli present (Fig. 5); bursa copulatrix divided into two sections by median constriction (bilobate) or not (Fig. 6), with or without additional “accessory pouch” situated near to spermatheca and accessory glands.

#### Species included

**(5).***Phylacastusancoralium* sp. nov., *P.crypticoides*, *P.makskacymirowi* sp. nov., *P.rhodesianus*, *P.striolatus*.

#### Distribution.

**Figure 7.**
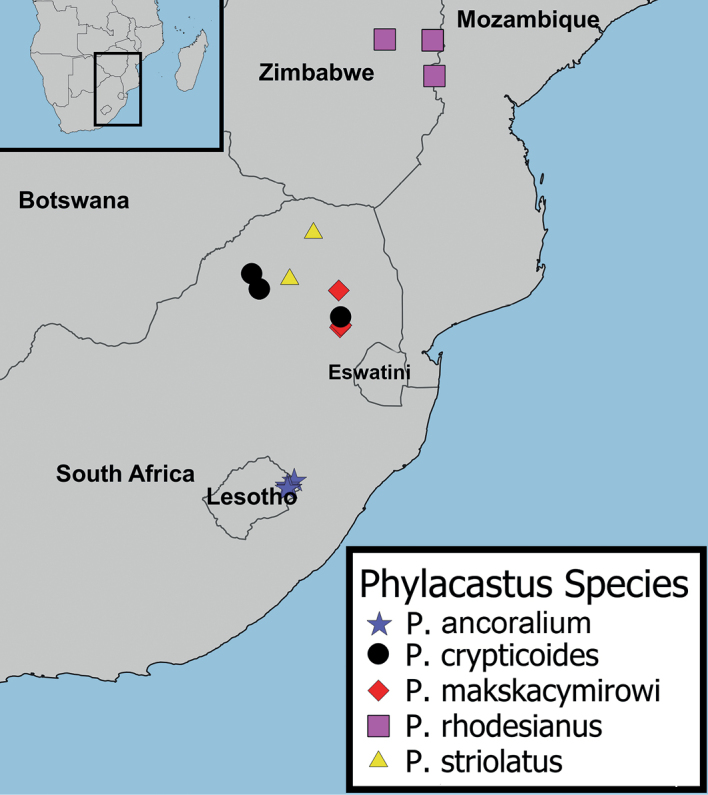
*Phylacastus* species distribution map. *P.ancoralium* (blue star), *Phylacastuscrypticoides* (black circle), *P.makskacymirowi* (red diamond), *P.rhodesianus* (pink square), *P.striolatus* (yellow triangle).

Southern Africa (Lesotho, South Africa, Zimbabwe) (Fig. 7).

### ﻿Key to the species of the genus *Phylacastus*

**Table:** 

1	Well-defined tubercles present on apical declivity of elytra (Fig. 2D)	**2**
–	Well-defined tubercles absent on apical declivity of elytra (Fig. 2B)	**3**
2	Male parameres widely spaced with large dorsal opening exposing median lobe (Fig. 4C); mentum parallel-sided and broad (Fig. 3C); elytral intervals densely punctate; generally larger (6–8 mm)	***P.rhodesianus* Fairmaire**
–	Male parameres not widely spaced, with small dorsal opening exposing at most only the tip of the median lobe (Fig. 4D); elytral intervals less densely punctate; mentum narrowing apically (Fig. 3B); generally smaller (4–6 mm)	***P.makskacymirowi* sp. nov.**
3	Aedeagus with ancorae (Fig. 4G); Ratio of ovipositor coxites I–IV to paraprocts nearly 1:1 (Fig. 5); elytra with at most weakly impressed striae on elytral disc, absent stria on apical declivity (Figs 2A, B)	***P.ancoralium* sp. nov.**
–	Aedeagus lacking ancorae (Fig. 4); Ratio of ovipositor coxites I–IV to paraprocts distinctly < 1:1 (Fig. 5); more clearly impressed elytral striae (Figs 2E, F)	**4**
4	Mentum with narrow carina/keel running up median (Fig. 3A); 5^th^ abdominal sulcus narrowly separated from apex (Fig. 3F)	***P.crypticoides* Koch**
–	Mentum lacking narrow carina/keel running up median; 5^th^ abdominal sulcus widely separated from apex (Fig. 3G)	***P.striolatus* Fairmaire**

### ﻿ Phylacastus ancoralium sp. nov.

Taxon classificationAnimaliaColeopteraTenebrionidae

82B62194-9DC8-5B1F-A38E-C462A5AE7DDA

https://zoobank.org/FDB06FBF-4FCA-4888-B36A-9E9724BDA235

Figs 1A , 2A, B , 3F , 4B, G , 5 , 6C

#### Material examined

**(data represents single specimens unless otherwise noted). *Holotype*** (TMNH): “S.Afr.;E. Lesotho Hodson’s Peak 300 m 29.37°S, 29.17°E; 11.3.1976;E-Y:1069 fr.und.stones, 3150 m leg. Endrödy-Younga.” With an additional label on red paper: “Holotype: *Phylacastusancoralium* Lumen & Kaminski”.

***Paratypes*** (*n* = 11) (TMNH and MIIZPAN): Two specimens with same data as Holotype (MIIZPAN). “S.Afr.Basutoland Makheke Mnts 15 miles ENE Mokhotlong. 8.IV.51 No. 268;Swedish South Africa Expedition 1950–1951; red label.” (MIIZPAN), “S.Afr., Lesotho Drakensbg,Black Mt. 29.31°S, 29.12°E; 9.3.1976;E-Y:1060 from under stones leg. Endrödy-Younga.”, “S.Afr.;E. Lesotho Hodson’s Peak 300 m 29.37°S, 29.17°E; 11.3.1976;E-Y:1067 from under stones leg. Endrödy-Younga” (five specimens)., “S.Afr., E.Lesotho Sani Pass Valley 29.39°S, 29.12°E; 10.3.1976; E-Y:1066 from under stones leg. Endrödy-Younga” (two specimens).

#### Diagnosis.

*Phylacastusancoralium* is highly modified compared with its congeners. In addition to its wide geographic separation from other species (Lesotho), it can be separated from all other species of *Phylacastus* via the elytra (with extremely weak to absent elytral striae), prosternum (weakly produced between forecoxae, rather than projecting more strongly beyond (Fig. 3E)), aedeagus with ancorae on the ventral surface of the parameres (Fig. 4G), and ovipositor relatively short compared to other species (ratio of ovipositor coxites I–IV to paraprocts nearly 1:1, rather than more distinctly < 1:1) (Fig. 5).

#### Etymology.

This species is named for the ancorae of the male aedeagus, which in Blaptinae are hypothesized to anchor the male genitalia during copulation. To date, this is the only species within the subtribe Eurynotina with ancorae.

#### Description.

Length 6–7 mm. ***Head***: punctures separated by ~1 feature diameter. Mentum midportion slightly narrowing apically, exposing lateral wings, midportion without distinct median carina. ***Prothorax***: pronotum finely punctate, punctures widely spaced, separated by > 1 feature diameter. Hypomeron lightly wrinkled and finely punctate. Prosternal process weakly produced between forecoxae. ***Pterothorax***: elytra width about equal to pronotal width. Elytral striae and intervals punctate; striae very weakly impressed or absent. Interval punctures fine and widely spaced (>1 feature diameter), distinctly smaller than strial punctures. Elytral tubercles absent. ***Abdomen***: ventrite V sulcus narrowly separated from apical border. ***Terminalia***: male: parameres tapering apically, fused basally with narrow opening at apex exposing median lobe. Each paramere bearing a small, ventral medial ancora. Female: Ratio of ovipositor coxites I–IV to paraprocts nearly 1:1. Bursa copulatrix not bilobate, accessory gland present near-to spermatheca, accessory pouch present.

#### Distribution.

Lesotho.

### ﻿ Phylacastus crypticoides

Taxon classificationAnimaliaColeopteraTenebrionidae

Koch

D597E3C9-67CA-5D0C-AC73-EB86C2AB54F9

**Figure 8.**
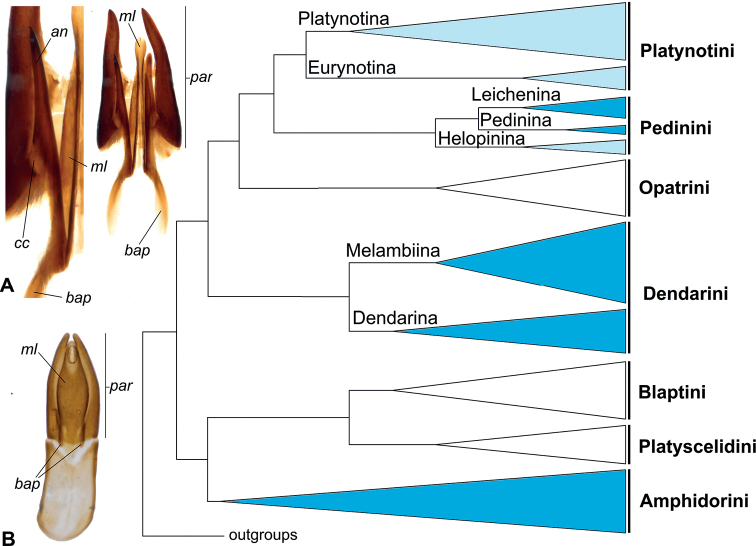
Distribution of *ancorae* in Blaptinae (displayed on Bayesian molecular topology from Kamiński et al. 2021a) **A***Heliopatesibericus* Mulsant & Rey (Dendarina) apical aedeagus **B***Phylacastuscrypticoides* aedeagus. Dark blue clades = all representatives have ancorae. Light blue clades = exceptions (with or without ancorae). White clades = no ancorae. Abbreviations: an - ancora, bap - basal apophysis, cc - sclerotized connection to parameres, ml - median lobe, par - parameres.

Figs 1C , 3A, D, F , 4E , 5 , 6B , 8B

Phylacastus crypticoides Koch, 1954a: 286. Kamiński 2016: 245.
= Phylacastuspretoriensis Koch, 1954a: 285, syn. nov. Kamiński 2016: 245.

#### Material examined

**(data represents single specimens unless otherwise noted). *Holotype*** (TMNH): “Lydenburg Distr. 1896 P.A. Krantz; Phylacastuscrypticoides DET.C.KOCH 1953; Holotype No: 1873 Phylacastuscrypticoides KOCH; crypticoides Koch; Eurynotus? sp..”

#### Additional material examined

**(TMNH).** “S.Afr.,N.Transvaal Nylsvley Met.Sta. 24.40°S, 28.42°E; 285.1975; E-Y:1160 humus, Berlese, open leg. Endrödy-Younga.”, “S.Afr.,N.Transvaal Nylsvley, Smith frm 24.40°S, 24.42°E 15.11.1975; E-Y: 952 cattle dung leg. Endrödy-Younga; trench; rep: 5 cage mesh 9 mm 7 day aft.sett.”*, “S.Afr.,N.Transvaal Nylsvley Met.Sta. 24.40°S, 28.42°E; 29.3.1976; E-Y:1112 sifted litter, open leg. Endrödy-Younga.”, “S.Afr.;Limpopo Prov. Lindani Nat Res 1336 m 24.02°S, 28.23°E; 8.12.2005; E-Y:3687 single, bushveld leg.Gusmann, Müller.”, “S.Afr.,N.Transvaal Nylsvley, Smith frm 24.40°S, 24.42°E 8.1.1976; E-Y: 990 sifted litter. Endrödy-Younga”*, “S.Afr.Tvl.Waterbg Lapalala Wilderness 23.49°S, 20.17°E; 16.8.1975; E-Y:829 from under stones leg. Endrödy-Younga” (seven specimens)^1^.

#### Notes.

Koch described both *Phylacastuscrypticoides* and *P.pretoriensis* (1954a), differentiating them from the already described *P.striolatus* and his additional species *P.rhodesianus* based on the following: *P.pretoriensis* with a basal pronotal margin that is reduced medially, and *P.crypticoides* with a cariniform structure of the mentum and a more apically positioned sulcus on abdominal ventrite V. Upon investigation here, the margination of the pronotal base, while variable, appears to be consistently present in all species with no uniform reduction in restricted populations or collection events examined here. The sulcus of abdominal ventrite V is also consistent between specimens of both of Koch’s species. Furthermore, *P.crypticoides* and *P.pretoriensis* specimens compared with his type material bear the carina attributed to *P.crypticoides*. As such, we have decided here to synonymize the two species under *P.crypticoides*.

#### Redescription.

Length 6–7 mm. ***Head***: punctures separated by < 1 diameter. Mentum broad, lateral wings concealed, midportion with thin, distinct medial carina. ***Prothorax***: pronotum punctate, punctures closely spaced, separated by ≤ 1 diameter. Hypomeron lightly wrinkled to rugose. Prosternal process produced between forecoxae (Fig. 3D). ***Pterothorax***: elytra width about equal to pronotal width. Elytral striae, intervals punctate; striae clearly impressed. Interval punctures closely spaced (≤1 diameter), slightly smaller than strial punctures. Elytral tubercles absent; apical declivity with at most weak bumps or callosities (Figs 1C, 2E, F). ***Abdomen***: ventrite V sulcus narrowly separated from apical border. ***Terminalia***: male: parameres tapering apically, fused basally with narrow opening at apex exposing median lobe. Female: ovipositor slightly elongate (ratio of ovipositor coxites I–IV to paraprocts < 1:1). Bursa copulatrix not bilobate, accessory gland present near-to spermatheca, accessory pouch absent.

#### Distribution.

South Africa.

### ﻿ Phylacastus makskacymirowi sp. nov.

Taxon classificationAnimaliaColeopteraTenebrionidae

CBD12F6A-3433-541E-AA68-FE32E5312910

https://zoobank.org/D45D3B72-5E44-451F-96B1-35D28A05E555

Figs 1B , 3B , 4D, F

#### Material examined

**(data represents single specimens unless otherwise noted). *Holotype*** (TMNH): “S.Afr.,E.Transvaal Berlin;Karst plat. 25.31°S, 30.46°E; 20.9.1986; E-Y:2279 groundtraps, 33 days leg. Endrödy-Younga; ground trap with meat bait.” With an additional label on red paper: “Holotype: *Phylacastusmakskacymirowi* Lumen & Kaminski”

***Paratypes*** (*n* = 11) (TMNH and MIIZPAN): Three additional specimens with same data as holotype (MIIZPAN). “S.Afr.,E.Transvaal Berlin;Karst plat. 25.31°S, 30.46°E; 23.10.1986; E-Y:2001 groundtraps, 42 days leg. Endrödy-Younga; ground trap with meat bait.”, “S.Afr.,E.Transvaal Berlin;Karst plat. 25.31°S, 30.46°E; 4.2.1986 E-Y:2414 under fungous logs leg. Endrödy-Younga.”, “S.Afr.; Mpumalanga 10 km E Kaapsehoop 25.36°S, 30.43°E; 4–6.1.2014: E-Y:3943 sifting; indigenous forest leg. Ruth Müller.”, “S.Afr.;Mpumalanga Sjonajona, Badplaas 24.44°S, 30.40°E; 11.11.2002; E-Y:3565 general collect. 1410 m leg. TMSA staff” (four specimens), “S.Afr.,E.Transvaal Berlin;Karst plat. 25.31°S, 30.46°E; 8.12.1986 E-Y:2363 fungous Pinus logs leg. Endrödy-Younga.”

#### Diagnosis.

As of this revision, this is the smallest species of the genus (4–6 mm). In addition to its size, this species is further defined by the presence of well-defined tubercles on the apical declivity of the elytra—a trait shared only by *P.rhodesianus*, which is larger and can be further differentiated by 1) punctures on elytral intervals (more numerous and dense in *P.rhodesianus*); 2) the shape of the mentum is broad, not tapered, further concealing the lateral wings in *P.rhodesianus* (Fig. 3C), tapers apically, exposing lateral wings in *P.makskacymirowi* (Fig. 3B); 3) aedeagus with a wide space between parameres, exposing large portion of median lobe in P.rhodesianus (Fig. 4C), narrow exposing only the tip of the median lobe in *P.makskacymirowi* (Fig. 4D).

#### Etymology.

Named after young bug enthusiast Maksymilian Jan Kacymirow (born on December 17, 2014 in Warsaw, Poland).

#### Description.

Length 4–6 mm. ***Head***: punctures separated by < 1 diameter. Mentum midportion medially raised but without distinct median carina, laterally tapering slightly toward apex, lateral wings exposed. ***Prothorax***: pronotum finely punctate, punctures smaller and widely spaced, separated by > 1 diameter. Hypomeron very finely punctate and lightly sculptured/wrinkled. Prosternal process produced between forecoxae. ***Pterothorax***: elytra wider than pronotal width. Elytral striae and intervals punctate; striae clearly impressed. Interval punctures fine, widely spaced (>1 diameter), distinctly smaller than strial punctures. Elytra distinctly tuberculate on apical declivity. ***Abdomen***: ventrite V sulcus narrowly separated from apical border. ***Terminalia***: male: parameres tapering apically, fused basally with narrow opening at apex exposing median lobe. Each paramere bearing a small, weak, subapical suture (Fig. 4F). Female: ovipositor slightly elongate (ratio of ovipositor coxites I–IV to paraprocts < 1:1). Bursa copulatrix not bilobate, accessory gland present near-to spermatheca, accessory pouch absent.

#### Distribution.

South Africa.

### ﻿ Phylacastus rhodesianus

Taxon classificationAnimaliaColeopteraTenebrionidae

Koch

C03E7BD9-0A9A-5A12-95A2-0CDB4AF88481

Figs 1D , 2C, D , 3C , 4C

Phylacastus rhodesianus Koch, 1954a: 287. Kamiński 2016: 245.

#### Material examined

**(data represents single specimens unless otherwise noted). *Holotype*** (TMNH): “Marandella Mashld XI.97 GKMarshall; Holotype No: 1877 Phylacastusrhodesianus KOCH; Phylacastusrhodesianus Koch DET.C.KOCH; rhodesianus Koch.”

#### Additional material examined

**(MNHN).** “9.VI.1970 Vumba SUD RHODESIE Cl. Besnard leg. 8.VI.1970 Inyanga SUD RHODESIE Cl. Besnard leg.” (10 specimens).

#### Redescription.

Length 6–8 mm. ***Head***: punctures separated by ≤ 1 diameter. Mentum midportion broad, concealing lateral wings, midportion without distinct median carina. ***Prothorax***: pronotum punctate, punctures closely spaced, separated by ~1 diameter. Hypomeron very lightly textured, without clear punctation. Prosternal process produced between forecoxae. ***Pterothorax***: elytra width about equal to pronotal width. Elytral striae and intervals punctate; striae impressed. Interval punctures fine, closely spaced (~1 diameter), distinctly smaller than strial punctures. Elytral tubercles present on apical declivity. ***Abdomen***: ventrite V sulcus narrowly separated from apical border. ***Terminalia***: male: parameres converging apically, fused basally with deep and wide opening at apex exposing median lobe (Fig. 4C). Female: ovipositor slightly elongate (ratio of ovipositor coxites I–IV to paraprocts < 1:1). Bursa copulatrix bilobate, accessory gland present near-to spermatheca, accessory pouch absent.

#### Distribution.

Zimbabwe.

### ﻿ Phylacastus striolatus

Taxon classificationAnimaliaColeopteraTenebrionidae

Fairmaire

A3B6CE66-76A3-565F-B021-A29E67B8BBF0

Figs 1E , 2E, F , 3G , 4A, H , 6A

Phylacastus striolatus Fairmaire, 1897: 117. Koch 1954a: 287; 1954b: 2; Kamiński 2016: 245.

#### Material examined

**(data represents single specimens unless otherwise noted).** Lectotype (MNHN) here designated: “Makapan (TR.) E. Simon 1893; Phylacastusstriolatus ? Cafrar?”. With an additional label on red paper: “Lectotype: *Phylacastusstriolatus* Fairmaire” Paralectotype (MNHN): single specimen with same data as lectotype.

#### Additional material examined

**(MIIZPAN).** “Transvaal Soutpansberg Mphome Magd Knothe S” (two specimens).

#### Redescription.

Length 8 mm. ***Head***: punctures separated by < 1 diameter. Mentum midportion broad, concealing lateral wings, midportion without distinct median carina. ***Prothorax***: pronotum punctate, punctures closely spaced, separated by ≤ 1 diameter. Hypomeron lightly wrinkled. Prosternal process produced between forecoxae. ***Pterothorax***: elytra width slightly greater than pronotal width. Elytral striae and intervals punctate; striae impressed. Interval punctures closely spaced (~1 diameter), smaller than strial punctures. Elytral tubercles absent; apical declivity with at most weak bumps or callosities. ***Abdomen***: ventrite V sulcus widely separated from apical border. ***Terminalia***: male: parameres converging apically, fused basally with small opening at apex exposing median lobe. Female: ovipositor slightly elongate (ratio of ovipositor coxites I–IV to paraprocts < 1:1). Bursa copulatrix bilobate, accessory gland present near-to spermatheca, accessory pouch present.

#### Distribution.

South Africa.

#### Note.

While Fairmaire did not specify the number of specimens he examined in his original description, he did make mention of the collector (E. Simon) and locality, making specimens of his syntype series identifiable. Two specimens from MNHN are here designated as the lectotypes to fix the taxonomic status of the species.

## ﻿Discussion

### ﻿Revision of genus *Phylacastus*

Overall, there were relatively few specimens available for study (*n* = 45), which may represent restricted ranges or collecting bias, although the collections we sampled represent older historical collections of their range. Despite the number of specimens, we borrowed and examined all of the type material, as well as additional representatives of all species. As of this revision, many of the traits that Koch (1954a) used to diagnose *Phylacastus* are still supported; however, some characters (e.g. the joining of the pronotum and elytra and the dilated male protarsi) were difficult to reliably confirm in the material gathered for this study. We interpret Koch’s (1954a) species *P.crypticoides* and *P.pretoriensis* as synonymous, as the traits used to differentiate them (mentum with sharp median carina in *P.crypticoides* and lack of basal pronotal margination in *P.pretoriensis*) were actually congruent between Koch’s type material for both species in the case of the mentum, and inconsistent throughout all the available material in the case of the pronotal margins. As to Koch’s (1954a) asserted relationship between *Phylacastus* and *Eurynotus*, additional phylogenetic study using morphological and/or molecular data will be required (Lumen and Kaminski in prep.). Currently, as of this revision their affiliation is not rejected—both genera have angled basal margins of the pronotum, angular prosternal processes, and tubercles on the apical declivity of the elytra (though often reduced in *Phylacastus*). The ovipositor of *Phylacastus* is only diagnostic for one species (*P.ancoralium*), and the genus appears to be overall congruent with other representatives of the subtribe (e.g. *Oncotus*), while also differing from *Eurynotus*, which has extremely long paraprocts (Iwan 2000; Banaszkiewicz 2006). There is some variation in the construction of the internal female anatomy of *Phylacastus*. In particular, *P.striolatus* and *P.rhodesianus* have a bursa copulatrix which is divided into two “lobes” by a median constriction (Fig. 6A), and there is an additional pouch situated near the spermatheca and accessory glands in *P.striolatus* and *P.ancoralium* (Fig. 6A, C). While the function of these structures is unclear at present, there may be similar structures in other representatives of the subtribe (e.g. *Eurynotuscapensis* (Fabricius) appears to have a similarly divided bursa copulatrix; Tschinkel 1978: fig. 1), which may be helpful for diagnosing groups or for phylogenetic inference. Additionally, there were some accessory structures on the aedeagi of *P.ancoralium* and *P.makskacymirowi.* Namely, the former possesses structures historically referred to as “lacinia” or “clavae”, and *P.makskacymirowi* has small, preapical sutures or grooves on the ventral side of the parameres. While the case of *P.ancoralium* is discussed in the below section, it is possible that the structures in *P.makskacymirowi* offer additional flexibility in the parameres.

### ﻿Male terminalia analyses

Our discovery of accessory structures on the parameres of *P.ancoralium* (Fig. 4G) raise questions not only on the phylogenetic placement of the species, but on the concept of Eurynotina and the way such structures have been defined historically in Tenebrionidae (e.g. Koch 1954a, b, 1955, 1956; Iwan 2001, 2002, 2004). The revelation of these structures highlights the necessity of investigating Eurynotina, as well as other enigmatic and poorly understood groups. One such subtribe, Helopinina Lacordaire (Pedinini Eschscholtz), is morphologically similar to Eurynotina, despite molecular evidence separating them (Kamiński et al 2021a, b; Fig. 8). In the case of Helopinina, there is also a marked reduction in accessory structures (similar to Eurynotina), though they can be differentiated in other ways (e.g. scale-like setation, non-reduced or elongate basal apophyses, basal versus apical tegmen length ratio, lack of stridulatory gula). A literary review revealed a myriad of terms used to refer to accessory structures associated with the median lobe, parameres, and tegmen (Antoine 1930; Español 1945; Doyen and Tschinkel 1982; Doyen 1984; Iwan 2001, 2002, 2004; Kamiński et al. 2019). Terms which have garnered the most use historically and recently are “clavae” and “lacinia.” Unfortunately, they have not been used uniformly, nor explicitly/formally defined in a way that is easily traceable or consistent. In fact, the two most used terms appear to follow authorship in North America (“clavae”—see Doyen and Tschinkel 1982; Doyen 1984; Aalbu et al. 2012; Johnston 2019) versus elsewhere (“lacinia”—see Español 1945; Iwan 2001). Thus far, the terms appear to have been used in an effort to qualitatively describe their shape. However, “clavae” is misleading in this regard and is much more widely used to refer to antennae (e.g. clava in Hymenoptera, Yoder et al. 2010). Additionally, while lacinia may adequately describe the form in some taxa, it misses the mark in others (e.g. *Anomalipus* spp.) and overlaps with much more widely used anatomical features (lacinia of the maxillary mouthparts of insects; Lawrence et al. 2011). Iwan (2004) gave a definition using the term lacinia (accessory spike- or hook-like structures which connect the median lobe with the inflexed alae of the apical piece), while also outlining their potential function (a means for the male to anchor itself internally during copulation as they extend/evert)—as well as the change in aedeagal function in groups which lacked them, such as Eurynotina (switching from lateral movement of “lacini” to a dorsoventral motion with a sclerotized median lobe and flexible parameres).

**Figure 9.**
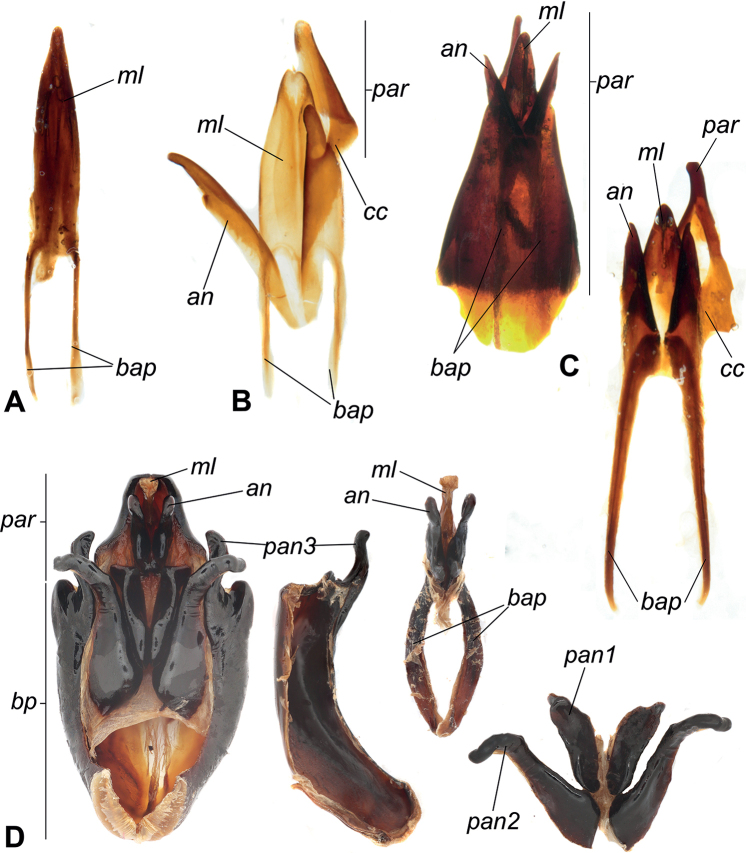
Dissections of ancorae variation and aedeagal morphology from Blaptinae**A***Amatodes* Dejean (Pedinini, Helopinina) median lobe with basal apophyses **B***Trigonopussimilis* Iwan (Platynotini, Platynotina) parameres, median lobe, and ancorae **C***Eleodesobscura* (Say) (Amphidorini) intact and extracted parameres, median lobe, and ancorae **D***Anomalipusmastodon* Fåhraeus, 1870 (Platynotini, Platynotina). Abbreviations: an - ancorae, bap - basal aphophyses, bp - basal portion of tegmen, cc - cuticular connection of ancorae to parameres, ml - median lobe, pan 1–3 - pseudo ancorae, par - parameres.

The aforementioned accessory structures to the median lobe and parameres have been recorded in two subfamilies and appear to be uncommon within Tenebrionidae. The first subfamily, Blaptinae, has several tribes (Amphidorini LeConte, Dendarini Mulsant & Rey, Pedinini, and Platynotini), and the second, Diaperinae, has one subtribe (Adelinina LeConte) that seem to have evolved variations of this characteristic morphology (Doyen 1984; Kamiński 2015b; Johnston 2019; Kamiński et al. 2021a). As a result of their unique and varied appearance, “clavae” or “lacinia” have been used to diagnose many tribes and subtribes (see Koch 1958; Doyen 1984; Iwan 2001); though in the case of some subtribes there are representatives that stand out contrastingly with their cohort as either having these structures (e.g. *Phylacastusancoralium*, unusual in Eurynotina; Fig. 4G) or lacking them (e.g. *Anomalipusheraldicus* Gerstaecker and *Anchophthalmus* spp. of Platynotina or *Amatodes* Dejean (Fig. 9A), *Ametrocera* Fåhraeus, and *Oncopteryx* Gebien of Helopinina).

**Figure 10.**
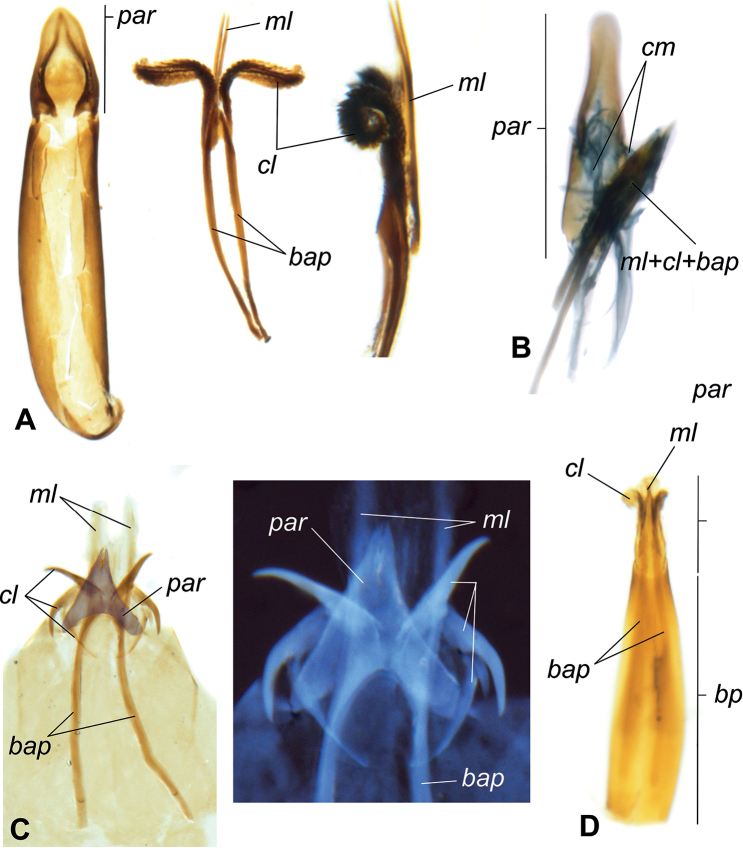
Sampled Adelenina (Diaperinae: Diaperini: Adelinina) aedeagi **A***Sitophagushololeptoides* (Laporte) **B***Adelina plana* (Fabricius) **C***Alphitophagusbifasciatus* (Say) **D***Gnatoceruscornutus* (Fabricius) Abbreviations: *bap* - basal apophyses, *cl* - “clavae”, *ml* - median lobe, *par* - parameres, *bp* - basal portion of aedeagus, *cm* - connective membrane.

We examined published records and dissected representatives of Blaptinae (e.g. *Anomalipus* and *Eleodes*) (Fig. 9C, D) to first solidify an anatomical definition for our accessory aedeagal structures of interest. Our dissections reveal these structures always mediate the connection between the parameres and median lobe in some capacity, though the diversity of morphological structures may obfuscate connecting points, giving the illusion they are linked only to the median lobe (Figs 8, 9). Additionally, even in less-closely related taxa, the conglomerate structure of the parameres and median lobe (plus accouterments) possess a median extension connected/merged with the basal apophyses (Fig. 9B–D), giving evidence for homology. To make referring to these structures more uniform, while also making their function more apparent, we propose naming these structures ancorae (singular: ancora) from the Latin *ancor*—in reference to the organ’s apparent reproductive function in anchoring the male to the female. We also hope that coining a new name for this feature will provide a means to better investigate homology, evolutionary strategies, and phylogeny. Our definition aims to unify the terminology and enable verification of homology in problematic cases. For example, some species of *Anomalipus* are known to possess several appendages of the tegmen (Endrödy-Younga 1988). Dissections of *Anomalipusmastodon* Fåhraeus (Fig. 9D) revealed most of these appendages are not linked to the median lobe or parameres; therefore, they cannot be regarded as ancorae. All of the extra appendages originate either from the basal piece of the tegmen (Fig. 9D, *pan3*) or are loosely attached by connecting membranes (Fig. 9D, *pan1* and *pan2*). Using the following criterion: connection to the parameres and the median lobe and linkage to the basal apopyses, we conclude that *A.mastodon* possesses only one pair of ancorae homological with the structures in other Platynotina (e.g. Fig. 9B). In another case, the subtribe Adelinina (Diaperinae: Diaperini) is defined by structures coined by Doyen (1984) as “clavae.” To test our definition, we also dissected representatives of *Adelina*, *Alphitophagus*, *Gnatocerus*, and *Sitophagus*. While all three possess accessory structures related to the median lobe and apex (parameres) of the aedeagus, there are several differences in comparison with what we observe in Blaptinae: 1) the median lobe is divided into two halves (Fig. 10), rather than fused as in Blaptinae (Figs 4, 8, 9); 2) the “clavae” are strongly connected with the basal aphophyses, which were long in all dissected specimens, but very weakly attached/associated with the median lobe (Fig. 10A); in Blaptinae all three structures are strongly associated/fused into a conglomerate structure (Figs 4, 8, 9); 3) the connection of the “clavae” to the parameres appears to be mediated by membranous structures (Fig. 10B). All the Blaptinae we observed have a much more strongly sclerotized connection (Fig. 9B, C). As a result, we propose that while these structures may be similar in form and operate in similar function(s), they do not fit our definition of ancorae focused on Blaptinae in particular. Diaperini Latreille as a tribe is very distantly related to Blaptinae phylogenetically (Kergoat et al. 2014; Kamiński et al. 2021a), and so these structures are likely not homologous, and likely would require additional examination in the future, and potential new terminology of their own. As such, we leave further investigation to other researchers focused on this and other more closely related groups.

## Supplementary Material

XML Treatment for Phylacastus

XML Treatment for Phylacastus ancoralium

XML Treatment for Phylacastus crypticoides

XML Treatment for Phylacastus makskacymirowi

XML Treatment for Phylacastus rhodesianus

XML Treatment for Phylacastus striolatus

## Footnotes

Some collecting events are likely erroneous in their coordinates (24.40°S, 24.42°E and 23.49°S, 20.17°E). These localities should be represented in northeastern South Africa (circa 24.40°S, 28.42°E); however, the coordinates as written on the labels refer to far-off localities in Botswana. As such, while the labels are recorded here, these points are omitted from the species’ range map.

## ﻿Appendix 1

**Table of *Phylacastus* distributional data in .csv format.**

Genus, Species,Verbatim Label, date (d.m.y), Determined Lat, Determined Long, note(s)

*Phylacastus*, *striolatus*, Makapan (TR.) E. Simon 1893; *Phylacastusstriolatus* ? Cafrar; , 1893, -24.1586, 29.1769, Type Locality; Point based on Makapan valley archeological site near to Mokopan.

*Phylacastus*, *striolatus*, Makapan (TR.) E. Simon 1893, 1893, -24.1586, 29.1769, Point based on Makapan valley archeological site near to Mokopan.

*Phylacastus*, *striolatus*, Transvaal Soutpansberg Mphome Magd Knothe S, -,-23.0084, 29.7690, point based on Soutpansberg Mountain.

*Phylacastus*, *striolatus*, Transvaal Soutpansberg Mphome Magd Knothe S, -,-23.0084, 29.7690, point based on Soutpansberg Mountain.

*Phylacastus*, *rhodesianus*, Marandella Mashld XI.97 GKMarshall; Holotype No: 1877 *Phylacastusrhodesianus* KOCH; *Phylacastusrhodesianus* Koch DET.C.KOCH; *rhodesianus* Koch, 11.1897, -18.1897, 31.5467, Type locality; Marondera (Marandella synonym).

*Phylacastus*, *rhodesianus*, 9.VI.1970 Vumba SUD RHODESIE Cl. Besnard leg.,9.VI.1970, -19.1000, 32.7833, Point based on Bvumba Mts.

*Phylacastus*, *rhodesianus*, 8.VI.1970 Inyanga SUD RHODESIE Cl. Besnard leg.,8.VI.1970, -18.2100, 32.7400,"Point based on Nyanga, Zimbabwe".

*Phylacastus*, *rhodesianus*, 8.VI.1970 Inyanga SUD RHODESIE Cl. Besnard leg.,8.VI.1970, -18.2100, 32.7400,"Point based on Nyanga, Zimbabwe".

*Phylacastus*, *rhodesianus*, 8.VI.1970 Inyanga SUD RHODESIE Cl. Besnard leg.,8.VI.1970, -18.2100, 32.7400,"Point based on Nyanga, Zimbabwe".

*Phylacastus*, *rhodesianus*, 8.VI.1970 Inyanga SUD RHODESIE Cl. Besnard leg.,8.VI.1970, -18.2100, 32.7400,"Point based on Nyanga, Zimbabwe".

*Phylacastus*, *rhodesianus*, 8.VI.1970 Inyanga SUD RHODESIE Cl. Besnard leg.,8.VI.1970, -18.2100, 32.7400,"Point based on Nyanga, Zimbabwe".

*Phylacastus*, *rhodesianus*, 8.VI.1970 Inyanga SUD RHODESIE Cl. Besnard leg.,8.VI.1970, -18.2100, 32.7400,"Point based on Nyanga, Zimbabwe".

*Phylacastus*, *rhodesianus*, 8.VI.1970 Inyanga SUD RHODESIE Cl. Besnard leg.,8.VI.1970, -18.2100, 32.7400,"Point based on Nyanga, Zimbabwe".

*Phylacastus*, *rhodesianus*, 8.VI.1970 Inyanga SUD RHODESIE Cl. Besnard leg.,8.VI.1970, -18.2100, 32.7400,"Point based on Nyanga, Zimbabwe".

*Phylacastus*, *rhodesianus*, 8.VI.1970 Inyanga SUD RHODESIE Cl. Besnard leg.,8.VI.1970, -18.2100, 32.7400,"Point based on Nyanga, Zimbabwe".

*Phylacastus*, *rhodesianus*, 8.VI.1970 Inyanga SUD RHODESIE Cl. Besnard leg.,8.VI.1970, -18.2100, 32.7400,"Point based on Nyanga, Zimbabwe".

*Phylacastus*, *makskacymirowi*, "S.Afr.,E.Transvaal Berlin;Karst plat. 25.31 S - 30.46 E; 23.10.1986; E-Y:2001 groundtraps, 42 days leg. Endrödy-Younga; ground trap with meat bait",23.10.1986, -25.52, 30.77.

*Phylacastus*, *makskacymirowi*, "S.Afr.,E.Transvaal Berlin;Karst plat. 25.31 S - 30.46 E; 20.9.1986; E-Y:2279 groundtraps, 33 days leg. Endrödy-Younga; ground trap with meat bait",20.8.1986, -25.52, 30.77, Type locality.

*Phylacastus*, *makskacymirowi*, "S.Afr.,E.Transvaal Berlin;Karst plat. 25.31 S - 30.46 E; 20.9.1986; E-Y:2279 groundtraps, 33 days leg. Endrödy-Younga; ground trap with meat bait",20.8.1986, -25.52, 30.77.

*Phylacastus*, *makskacymirowi*, "S.Afr.,E.Transvaal Berlin;Karst plat. 25.31 S - 30.46 E; 20.9.1986; E-Y:2279 groundtraps, 33 days leg. Endrödy-Younga; ground trap with meat bait",20.8.1986, -25.52, 30.77.

*Phylacastus*, *makskacymirowi*, "S.Afr.,E.Transvaal Berlin;Karst plat. 25.31 S - 30.46 E; 20.9.1986; E-Y:2279 groundtraps, 33 days leg. Endrödy-Younga; ground trap with meat bait",20.8.1986, -25.52, 30.77.

*Phylacastus*, *makskacymirowi*, "S.Afr.,E.Transvaal Berlin;Karst plat. 25.31 S - 30.46 E; 4.2.1986 E-Y:2414 under fungous logs leg. Endrödy-Younga",4.2.1986, -25.52, 30.77.

*Phylacastus*, *makskacymirowi*, "S.Afr.,E.Transvaal Berlin;Karst plat. 25.31 S - 30.46 E; 8.12.1986 E-Y:2363 fungous Pinus logs leg. Endrödy-Younga",8.12.1986, -25.52, 30.77.

*Phylacastus*, *makskacymirowi*, S.Afr.;Mpumalanga 10km E Kaapsehoop 25.36 S - 30.43 E; 4-6.1.2014: E-Y:3943 sifting; indigenous forest leg. Ruth Müller, 4-6.1.2014, -25.60, 30.72.

*Phylacastus*, *makskacymirowi*, "S.Afr.;Mpumalanga Sjonajona, Badplaas 24.44 S - 30.40 E; 11.11.2002; E-Y:3565 general collect. 1410m leg. TMSA staff",11.11.2002, -25.73, 30.67.

*Phylacastus*, *makskacymirowi*, "S.Afr.;Mpumalanga Sjonajona, Badplaas 24.44 S - 30.40 E; 11.11.2002; E-Y:3565 general collect. 1410m leg. TMSA staff",11.11.2002, -25.73, 30.67.

*Phylacastus*, *makskacymirowi*, "S.Afr.;Mpumalanga Sjonajona, Badplaas 24.44 S - 30.40 E; 11.11.2002; E-Y:3565 general collect. 1410m leg. TMSA staff",11.11.2002, -25.73, 30.67.

*Phylacastus*, *makskacymirowi*, "S.Afr.;Mpumalanga Sjonajona, Badplaas 24.44 S - 30.40 E; 11.11.2002; E-Y:3565 general collect. 1410m leg. TMSA staff",11.11.2002, -25.73, 30.67.

*Phylacastus*, *crypticoides*, Lydenburg Distr. 1896 P.A. Krantz; *Phylacastuscrypticoides* DET.C.KOCH 1953; Holotype No: 1873 *Phylacastuscrypticoides* KOCH; *crypticoides* Koch; Eurynotus? sp.,1896, -25.0960, 30.4460, Approximated in Google Earth.

*Phylacastus*, *crypticoides*, "S.Afr.,N.Transvaal Nylsvley Met.Sta. 24.40 S - 28.42 E; 285.1975; E-Y:1160 humus, Berlese, open leg. Endrödy-Younga",28.5.1975, -25.67, 28.70.

*Phylacastus*, *crypticoides*, "S.Afr.,N.Transvaal Nylsvley, Smith frm 24.40S-24.42E 15.11.1975; E-Y: 952 cattle dung leg. Endrödy-Younga; trench; rep: 5 cage mesh 9mm 7 day aft.sett.",15.11.1975, -25.67, 28.70.

*Phylacastus*, *crypticoides*, "S.Afr.,N.Transvaal Nylsvley Met.Sta. 24.40 S - 28.42 E; 29.3.1976; E-Y:1112 sifted litter, open leg. Endrödy-Younga",29.3.1976, -25.67, 28.70.

*Phylacastus*, *crypticoides*, "S.Afr.;Limpopo Prov. Lindani Nat Res 1336m 24.02 S - 28.23 E; 8.12.2005; E-Y:3687 single, bushveld leg.Gusmann, Müller",8.12.2005, -24.03, 28.38.

*Phylacastus*, *crypticoides*, "S.Afr.,N.Transvaal Nylsvley, Smith frm 24.40S-24.42E 8.1.1976; E-Y: 990 sifted litter. Endrödy-Younga",8.1.1976, -25.67, 28.70.

*Phylacastus*, *crypticoides*, S.Afr.Tvl.Waterbg Lapalala Wilderness 23.49 S- 20.17 E; 16.8.1975; E-Y:829 from under stones leg. Endrödy-Younga, 16.8.1975, -23.82, 20.28.

*Phylacastus*, *crypticoides*, S.Afr.Tvl.Waterbg Lapalala Wilderness 23.49 S- 20.17 E; 16.8.1975; E-Y:829 from under stones leg. Endrödy-Younga, 16.8.1975, -23.82, 20.28.

*Phylacastus*, *crypticoides*, S.Afr.Tvl.Waterbg Lapalala Wilderness 23.49 S- 20.17 E; 16.8.1975; E-Y:829 from under stones leg. Endrödy-Younga, 16.8.1975, -23.82, 20.28.

*Phylacastus*, *crypticoides*, S.Afr.Tvl.Waterbg Lapalala Wilderness 23.49 S- 20.17 E; 16.8.1975; E-Y:829 from under stones leg. Endrödy-Younga, 16.8.1975, -23.82, 20.28.

*Phylacastus*, *crypticoides*, S.Afr.Tvl.Waterbg Lapalala Wilderness 23.49 S- 20.17 E; 16.8.1975; E-Y:829 from under stones leg. Endrödy-Younga, 16.8.1975, -23.82, 20.28.

*Phylacastus*, *crypticoides*, S.Afr.Tvl.Waterbg Lapalala Wilderness 23.49 S- 20.17 E; 16.8.1975; E-Y:829 from under stones leg. Endrödy-Younga, 16.8.1975, -23.82, 20.28.

*Phylacastus*, *crypticoides*, S.Afr.Tvl.Waterbg Lapalala Wilderness 23.49 S- 20.17 E; 16.8.1975; E-Y:829 from under stones leg. Endrödy-Younga, 16.8.1975, -23.82, 20.28.

*Phylacastus*, *pseudoclavum*, S.Afr.Basutoland Makheke Mnts 15 miles ENE Mokhotlong. 8.IV.51 No. 268;Swedish South Africa Expedition 1950-1951; red label, 8.IV.1951, -29.19, 29.29, Approximated in Google Earth.

*Phylacastus*, *pseudoclavum*, "S.Afr.;E. Lesotho Hodson's Peak 300m 29.37S - 29.17 E; 11.3.1976;E-Y:1069 fr.und.stones, 3150m leg. Endrödy-Younga",11.3.1976, -29.62, 29.28, Type locality.

*Phylacastus*, *pseudoclavum*, "S.Afr.;E. Lesotho Hodson's Peak 300m 29.37S - 29.17 E; 11.3.1976;E-Y:1069 fr.und.stones, 3150m leg. Endrödy-Younga",11.3.1976, -29.62, 29.28.

*Phylacastus*, *pseudoclavum*, "S.Afr.;E. Lesotho Hodson's Peak 300m 29.37S - 29.17 E; 11.3.1976;E-Y:1069 fr.und.stones, 3150m leg. Endrödy-Younga",11.3.1976, -29.62, 29.28.

*Phylacastus*, *pseudoclavum*, S.Afr.;E. Lesotho Hodson's Peak 300m 29.37S - 29.17 E; 11.3.1976;E-Y:1067 from under stones leg. Endrödy-Younga, 11.3.1976, -29.62, 29.28.

*Phylacastus*, *pseudoclavum*, S.Afr.;E. Lesotho Hodson's Peak 300m 29.37S - 29.17 E; 11.3.1976;E-Y:1067 from under stones leg. Endrödy-Younga, 11.3.1976, -29.62, 29.28.

*Phylacastus*, *pseudoclavum*, S.Afr.;E. Lesotho Hodson's Peak 300m 29.37S - 29.17 E; 11.3.1976;E-Y:1067 from under stones leg. Endrödy-Younga, 11.3.1976, -29.62, 29.28.

*Phylacastus*, *pseudoclavum*, S.Afr.;E. Lesotho Hodson's Peak 300m 29.37S - 29.17 E; 11.3.1976;E-Y:1067 from under stones leg. Endrödy-Younga, 11.3.1976, -29.62, 29.28.

*Phylacastus*, *pseudoclavum*, S.Afr.;E. Lesotho Hodson's Peak 300m 29.37S - 29.17 E; 11.3.1976;E-Y:1067 from under stones leg. Endrödy-Younga, 11.3.1976, -29.62, 29.28.

*Phylacastus*, *pseudoclavum*, "S.Afr., Lesotho Drakensbg, Black Mt. 29.31 S - 29.12 E; 9.3.1976;E-Y:1060 from under stones leg. Endrödy-Younga",9.3.1976, -29.52, 29.20.

*Phylacastus*, *pseudoclavum*, "S.Afr., E.Lesotho Sani Pass Valley 29.39 S - 29.12 E; 10.3.1976; E-Y:1066 from under stones leg. Endrödy-Younga",10.3.1976, -29.52, 29.20.

*Phylacastus*, *pseudoclavum*, "S.Afr., E.Lesotho Sani Pass Valley 29.39 S - 29.12 E; 10.3.1976; E-Y:1066 from under stones leg. Endrödy-Younga",10.3.1976, -29.52, 29.20.
